# Detecting Fall Risk and Frailty in Elders with Inertial Motion Sensors: A Survey of Significant Gait Parameters

**DOI:** 10.3390/s21206918

**Published:** 2021-10-19

**Authors:** Luisa Ruiz-Ruiz, Antonio R. Jimenez, Guillermo Garcia-Villamil, Fernando Seco

**Affiliations:** Centre for Automation and Robotics (CAR), Consejo Superior de Investigaciones Científicas (CSIC)-UPM, Ctra. Campo Real km 0.2, La Poveda, Arganda del Rey, 28500 Madrid, Spain; antonio.jimenez@csic.es (A.R.J.); guillermo.gv@csic.es (G.G.-V.); fernando.seco@csic.es (F.S.)

**Keywords:** frailty, gait analysis, inertial sensor

## Abstract

In the elderly, geriatric problems such as the risk of fall or frailty are a challenge for society. Patients with frailty present difficulties in walking and higher fall risk. The use of sensors for gait analysis allows the detection of objective parameters related to these pathologies and to make an early diagnosis. Inertial Measurement Units (IMUs) are wearables that, due to their accuracy, portability, and low price, are an excellent option to analyze human gait parameters in health-monitoring applications. Many relevant gait parameters (e.g., step time, walking speed) are used to assess motor, or even cognitive, health problems in the elderly, but we perceived that there is not a full consensus on which parameters are the most significant to estimate the risk of fall and the frailty state. In this work, we analyzed the different IMU-based gait parameters proposed in the literature to assess frailty state (robust, prefrail, or frail) or fall risk. The aim was to collect the most significant gait parameters, measured from inertial sensors, able to discriminate between patient groups and to highlight those parameters that are not relevant or for which there is controversy among the examined works. For this purpose, a literature review of the studies published in recent years was carried out; apart from 10 previous relevant reviews using inertial and other sensing technologies, a total of 22 specific studies giving statistical significance values were analyzed. The results showed that the most significant parameters are double-support time, gait speed, stride time, step time, and the number of steps/day or walking percentage/day, for frailty diagnosis. In the case of fall risk detection, parameters related to trunk stability or movements are the most relevant. Although these results are important, the total number of works found was limited and most of them performed the significance statistics on subsets of all possible gait parameters; this fact highlights the need for new frailty studies using a more complete set of gait parameters.

## 1. Introduction

Medical and technological advances continue to improve the span and quality of life of the general population. According to the World Health Organization, the proportion of the world’s population over 60 y will double from 11% to 22% in 2050 [1]. Global aging is a triumph, but also a challenge for healthcare systems, because of physical decline and an intensification of chronic diseases associated with the elderly.

Frailty is a weakness syndrome (set of symptoms) associated with aging, which represents a physiological decline, i.e., a geriatric condition characterized by an increased vulnerability to external stressors. Its global prevalence is over 17%, and it increases with age and is greater in women than men [2]. Frailty is closely related to adverse events such as falls, decreasing mobility, hindrances in the activities of daily life, hospitalization, and deaths.

Fried’s phenotype is one of the most widely accepted and used criteria for frailty diagnosis [3]. It is based on five criteria or conditions, which are collected in the Table 1: (1) low physical activity (weekly exercise hours or calories), (2) slow walking speed (m/s), (3) poor endurance (feeling exhausted or having low energy), (4) weakness or low grip strength (kg), and (5) unintentional weight loss (change in kg). The existence of these conditions determines the state of the patient: robust, prefrail (one or two of Fried’s conditions) or frail (three or more conditions).

Traditionally, frailty has been diagnosed using the Fried criteria or other frailty scales or functional tests performed in the doctor’s office, such as gait speed test, the Timed Up and Go test (TUG) [4], or the Short Physical Performance Battery (SPPB) [5]. Currently, technological advances are providing more accurate gait analysis methods to detect frailty and fall risk in elderly people. Moreover, these methods allow more unbiased and realistic tests to be carried out (in the presence of a doctor, the patients tend to outperform) [6]. These advanced methods for gait analysis can be divided into wearable and nonwearable systems, and an overview of them is carried out in [7]. Nonwearable systems are based on image processing or floor sensors. Image processing systems are usually formed by an array of cameras that capture human movements; however, these systems can be made up of other types of optical sensors such as laser range scanners or infrared sensors. Floor sensors consist of a set of pressure sensors located on force platforms, which capture the human gait by measuring the force exerted by the subject’s feet during the gait. Alternatively, wearable systems are sensors located on different parts of the body, such as the feet, legs, back, or chest. There are several sensors: force sensors, which are located under the foot and measure the ground reaction force; electromyography, measuring the electric signal caused by contracting muscles with surface electrodes; inertial sensors or Inertial Measurement Units (IMUs), able to capture the acceleration and angular rate and from them estimate velocities, angles, or gait cycle ratios.

The advantages and disadvantages of wearable and nonwearable systems are determined by their characteristics and goal applications. Nonwearable systems are nonintrusive and highly precise and have a large measurement capacity; however, they are limited by a controlled environment, so tests must be carried out in laboratories, it being impossible to monitor real life outside, and have a high cost. Because of these reasons, wearable sensors are most commonly used in research, and some of their advantages are: the low cost and the possibility of measuring in any place without needing controlled environments. Their limitations must be taken into account: battery duration is limited, so very long time tests (e.g., several days) cannot be performed, and in some cases, complex algorithms to process the data and remove the noise effects (drifts) are needed.

A review that analyzed the available methodologies for gait phase detection [8] showed an increase in the use of IMUs due to their advantages over other type of sensors. There are several studies that supported the use of inertial sensors in medical gait analysis, representing a new approach that improves the classical methods. A comparison review between traditional systems and inertial sensors for gait analysis in healthy and pathological adults was carried out by [9]. It showed a good concordance between both systems and supported the use of inertial sensors as a portable solution for gait analysis in clinical and laboratory settings, as well as in outdoor spaces in real-life conditions, with good reliability.

Studying the relationship between frailty and gait analysis in the literature, we found 10 different reviews related to these topics. Table 2 contains a summary of these works, arranged by the following column fields: author, year, patient’s medical condition, gait analysis technology, number of papers (M) included in each review, and relevant conclusions in the review. Frailty is closely related to fall risk, so reviews that focused on fall risk in older people are also included in this table. Some reviews studied a large number of technologies [10,11,12,13,14,15], whereas [16,17,18] focused on the use of inertial sensors. These studies supported the use of gait analysis systems for the evaluation of frailty or fall risk in elderly patients; some of them identified a few gait parameters related to frailty status or fall risk, as the stride length, the double support time, the reduction of some parameters (cadence, gait velocity), or the gait variability, as well as the sensor locations, the lower trunk being the most common body location among the studies.

By analyzing these revisions, we detected a variety of parameters to be measured, with different sensor body locations and a large variety of patient studies. However, we did not find any review that analyzed in depth which are the most relevant parameters for the diagnosis of frailty using inertial sensors. We detected some limitations in these reviews with respect to our goal. Most of them did not include studies with frail patients [8,9,11,12,16,17,19,20], and those focused on frailty did not emphasize the use of inertial sensors or, specifically, the significance of gait parameters to identify which are the most relevant gait parameters related to frailty [10,13,21]. For these reason and to give a response to this problem, in this work, we carried out a review of the state-of-the-art that highlights the significance of gait parameters that others studies have already quantified using inertial sensors and present them in an integrated way, so that an engineer can use it as a guide to design a gait analysis tool with IMUs by estimating in advance the most promising parameters from inertial measurements to identify the frailty status and fall risk.

The paper is organized as follows: Section 2 gives the background knowledge on and definitions of frailty, what scoring tests are available, and the used gait cycle terminology (e.g., phase events and periods). You can skip this section if proficient in this field. Section 3 describes the methodology used and inclusion criteria for the literature search, selection, and data synthesis. Section 4 describes the results: a brief description of each study included, the tabulation of the most important characteristic of each study such as the number of sensors, location, or type of patients and the tabulation of the gait parameters and their significance. Section 5 contains the discussion, including some limitations detected, and finally, Section 6 ends the paper with the most relevant conclusions.

## 2. Background Concepts

This section details the concept of frailty and the classical methods for its diagnosis, as well as gait analysis, describing the gait cycle and the most important parameters that can be extracted from it.

### 2.1. Frailty Definition and Scoring Tests

Frailty is a syndrome (set of symptoms) associated with aging and represent a decline in different functional abilities [22]. According to Fried et al. [3], a frailty condition is met when three (or more) out of five energy-related criteria are satisfied, as shown in Table 1. Alternatively, frailty has been determined as a risk index by counting the number of deficits accumulated over time (termed “Frailty Index (FI)”) [22] including disability, diseases, physical and cognitive impairments, and geriatric syndromes (e.g., falls). However, the five-component phenotype proposed by Fried et al. [3] is easier to use and more practical in a clinical setting, compared to the FI that typically contains thirty to seventy items.

It is commonly accepted that there exist three frailty-related states: robust, prefrail (when one or two above-cited criteria are present), and frail (three or more conditions). Several studies for long periods (about 5 y) [22] showed that more than 50% of the elderly population in nursing homes had at least one transition between any two of the three frailty states. According to Fried et al. [3], in a study for 65+ y persons, 46% did not present any conditions, 32% 1 condition, 15% 2 conditions, and only 6%, 1%, and 0.2% for 3, 4, and 5 conditions simultaneously. The most frequent condition were, in order, low activity (22% of the cases), slow walk and grip strength (20% both), exhaustion (17%), and weight loss (6%).

The evolution of frailty is heterogeneous, complex, and unknown; there is evidence that a genetic basis exists; in addition, medical diseases and life habits determine its further development. The early detection of these functional changes or deficits at the physiologic level is important for preventing or reversing the development of frailty, before reaching disability.

Many frailty conditions are related to the motion of a person. From the five conditions established by Fried et al. [3] for frailty condition, three of them, low energy, slow waking speed, and low physical activity, could be measured with motion sensors (e.g., inertial). Weight loss and grip strength conditions would need to be assessed with other sensors. Regarding low grip strength, in most of the cases, it is a consequence of general weakness, so we could also assess it with a hand-held dynamometer, from the time needed to perform strength-related activities, such as getting up/down from a chair, which could also be measured from inertial sensors.

While relative motion is important, the absolute movement of a person within his/her living area is also relevant. The low physical activity can be measured by his/her spatial mobility, defined as the trajectory’s length that a person moves on purpose during daily routines, as well as the frequency of mobility within a specific time frame.

#### Frailty Scoring Tests

Walking speed is the most common objective test of functional limitation in the literature, and it is one of the components of Fried’s frailty phenotype. The speed threshold (cut point) with the best predictive value for frailty diagnosis is 0.8 m/s. The most commonly used tests are those that calculate the normal walking speed at traveled distances between 4 m and 6 m [23]. There are more complex walking tests, called dual-task tests, in which the patient walks while performing another task, such as counting from one to one-hundred. These dual-task tests are also used to detect cognitive decline or fall risk.

The *Timed Up and Go Test* (TUG) was specifically designed to quantify mobility and has proven to be a good predictor of deteriorating health status [4]. It contains a walking speed test, with a 180${}^{\circ }$ turn, in combination with an initial get up from a chair and a final sit down. Normal time is considered less than 10 s; times longer than 15 s are related to a high risk of falls; above 20 s is related to a high risk of disability.

Other tests focus on the action of sitting down and getting up from a chair. The *30-s Chair Stand Test* (30-s CST) [24] consists of counting how many times the patient is able to sit down and stand up for 30 s, whereas the *Five-Times Sit to Stand* (FTSS), quantifies the total time to do it five times [25].

The *Short Physical Performance Battery* (SPPB) [5] is one of the most validated and reliable tests for detecting frailty and predicting disability, in a scale from zero to twelve points. It is also known as the Guralnik test: (1) balance (with different foot positions); (2) speed (time in 4 m path); (3) sitting and standing five times (time needed). Each part is scored from 0–4, and the sum of the total score provides a classification: 0–3 = severe functional limitation (dependent subject), 4–6 = moderate functional limitation (subject with frailty), 7–9 = low functional limitation (prefrail subject), and 10–12 = absent functional limitation (robust subject).

### 2.2. Gait Analysis Definitions

Gait analysis is one of the most commonly used methods to detect frailty and fall risk in elderly people [16]. It consists of identifying the different phases of the gait cycle and extracting their characteristic parameters. As mentioned above, frailty is directly related to changes in gait, namely slower gait speed. As frailty increases, mobility capacity decreases and gait changes become more evident. Through gait analysis and quantification of the gait parameters, differences can be found between robust, prefrail, and frail patients. These differences allow discriminating between patient groups, to diagnose frailty, and to provide early medical attention.

The following is an explanation of the gait cycle phases/events and the parameters that can be extracted from sequences of gait cycles (e.g., variability or asymmetries).

#### 2.2.1. Gait Cycle Phases

**Figure 1:**
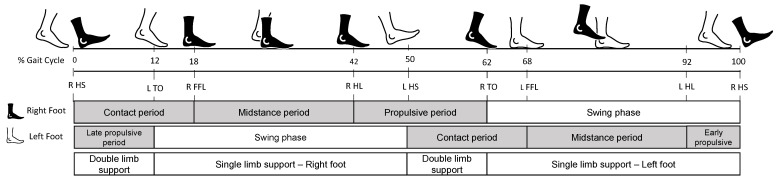
Human Gait Cycle (GC). The first line under the foot sketches represents the duration in percentage of the GC. Below the different gait events: Heel Strike (HL), Toe-Off (TO), Full Forefoot Load (FFL), Heel Lift (HL). Below are the segmented phases for the right and left feet. The last part indicates the relation between the left and right feet. The gait phase’s duration is approximate and varies between authors; these percentages are accepted in the literature.

The *Gait Cycle* (GC) is mainly divided into two phases: stance and swing, which usually last 60% and 40% of the GC, respectively. The stance phase occurs when the foot is in contact with the ground, while during the swing phase, the foot is in motion and without contact with the floor. The GC begins when the foot first contacts the ground with the heel (Heel Strike (HS)) and ends the next time the same foot contacts the ground with the heel again. The stance phase is subdivided into three phases: contact period (lasting 18% of the GC, from HS to Foot Flat (FFL)), midstance period (full foot on the ground, lasting 24% of the GC), and propulsive period (starting when the Heel Lifts (HL) and ending when the Toe is Off (TO), lasting about 20% of the GC). Figure 1 shows the gait cycle and the different phases. Single-limb support refers to the period when only one foot is in contact with the ground, whereas Double-Limb Support (DS) occurs when both feet are in contact with the ground simultaneously.

#### 2.2.2. Gait Parameters

**Figure 2:**
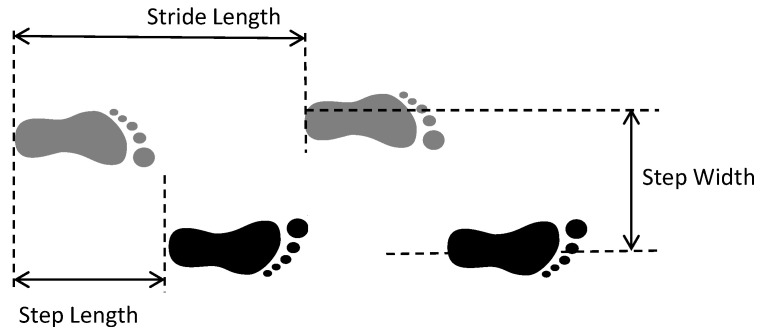
Important spatial gait parameters: step length, stride length, and step width. Black and grey shaded colors in footprints represent the right and left feet, respectively.

The most common spatiotemporal parameters that can be obtained from the identification of gait phases are detailed below:

- Step length (m): distance between the point of initial contact of one foot (HS) and the point of initial contact of the opposite foot (e.g., HS between the left and right feet);
- Stride Length (SL) (m): the distance between successive points of initial contact (HS) of the same foot;
- Step width (m): lateral separation between both feet. The differences among stride length, step length, and step width can be found in Figure 2;
- Step time (s): time between two consecutive heel strikes;
- Stride time (s) or Gait Cycle Time (GCT): time between two consecutive heel strikes by the same foot, as well as time needed to complete a full gait cycle;
- Gait speed (m/s): the stride length divided by the total GCT.
- Cadence (steps/min): number of steps in 1 min.

The duration of the different phases and periods of the gait are considered as temporal parameters: swing phase time, stance phase time, double-support time, or propulsive period time, are studied in the literature.

**Figure 3:**
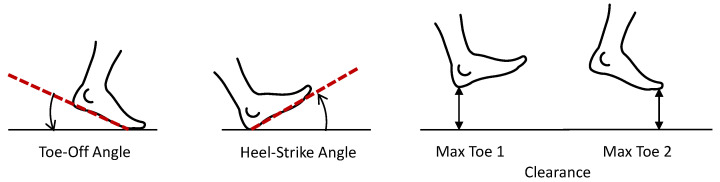
Specific spatial gait parameters: toe-off angle, heel strike angle and clearance.

In addition, there are other more specific spatial parameters, related to the position of the foot during a certain moment of the gait cycle, show Figure 3. In the case of inertial sensors, these parameters are only measurable when the sensor is placed on the foot:

- Toe-off angle (degrees): the degree of inclination of the foot at the moment of take-off;
- Heel strike angle (degrees): the degree of inclination of the foot at the moment when the heel touches the ground;
- Clearance, max toe (m): the maximum height that the foot reaches during swing phase.

Now that all these definitions and concepts are clear, we continue with the next fundamental sections.

## 3. Literature Research Methodology

### 3.1. Search Strategy and Eligibility Criteria

We conducted a literature search in major databases and search engines such as Google Scholar, PubMed, and Web of Science. The search topics were those related to gait analysis with inertial sensors for the diagnosis of frailty or fall risk. The combination of key words used was: gait AND (frail* OR fall risk OR faller OR falls) AND (inertial sensor OR wearable OR accel* OR gyro*) AND (old* OR elder* OR geriatric OR geront*). The results were limited to articles written in the English language and publication dates during the last 10 y. In addition, other relevant studies were identified from the list of references of each article. These studies were included provided that they satisfied the inclusion criteria:

- Older adult patients (≥60 y);
- Diseases: frailty or fall risk;
- The use of inertial sensors in gait analysis;
- The extraction of gait parameters from inertial data recorded during the walking, physical daily activity, and frailty assessment tests described;
- Discrimination between patients groups from gait parameters.

### 3.2. Data Extraction

The selected studies were individually analyzed in order to extract the most characteristic information of each work: author, year, patients’ information: medical condition (frailty, fall risk, and others), number of patients, and percentage of healthy patients included; sensor information: model, number of IMUs used, and location; performed tests. Gait parameters were extracted when authors identified a statistical significance between patient groups.

### 3.3. Data Synthesis

All these data are summarized and collated in tables. Lastly, the gait parameters extracted, used, and defined as relevant in each study were analyzed and collected in tables.

## 4. Results

This section includes the results obtained from the literature review, applying the methodology described in the previous section.

A total of 22 studies were included in the review, as shown in Table 3, which contains the most relevant information extracted from each article: author, year, patients’ information: medical condition (frailty, fall risk and others), number of patients and percentage of healthy patients included; sensor information: model, axis, number of IMUs used, and location; performed tests.

The third column, *IMUs and location*, contains information about the sensors and their locations, where the second row represents the number of axes (X Acc + Y Gyr) of the accelerometers and gyroscopes used in the study. Authors employed different inertial sensors with different characteristics. Some of them, as the Xsens models, have tri-axial accelerometers and gyroscopes, but also magnetometers. Others, as Shimmer or LEGSys models, have triaxial accelerometers and gyroscopes. Among the papers included, the use of sensors composed of tri-axial accelerometers such as PAMSYS, Axivity, or Dynaport was also common, with at least 11 papers using these devices. Some authors used the orthogonal tri-axial sensors, but other used a single axis aligned with the anteroposterior, mediolateral, or vertical plane.

On the other hand, the *Motion tests* column contains information about the type of test: walking, daily physical activity, or classic tests. The time duration or longitude of walking tests is also included.

### 4.1. Study Characteristics

The studies included had a different number and patient characteristics, as well as a different inertial system and motion test. The characteristics are summarized below.

#### 4.1.1. Patients

Patients included in the studies were older adults (over 60 y); the number of participant in each study varies from 17 to 718, the median being 121 patients per study. The percentage of healthy subjects included in the studies was from 23% to 62%, with a median value equal to 45%.

The studies included patients with frailty, fall risk, both, or other diseases: 11 studies were focused on patients with frailty [15,24,26,27,28,29,31,35,36,41,42], 6 studies patients with fall risk [6,18,30,33,37,39], and 2 studies combining frailty and fall risk: [25,38]. Two studies included patients with frailty plus mind and peripheral artery disease [32,40], respectively; and one study [34] included patients with fall risk and Parkinson’s.

#### 4.1.2. Inertial Sensor: Number and Location

**Figure 4:**
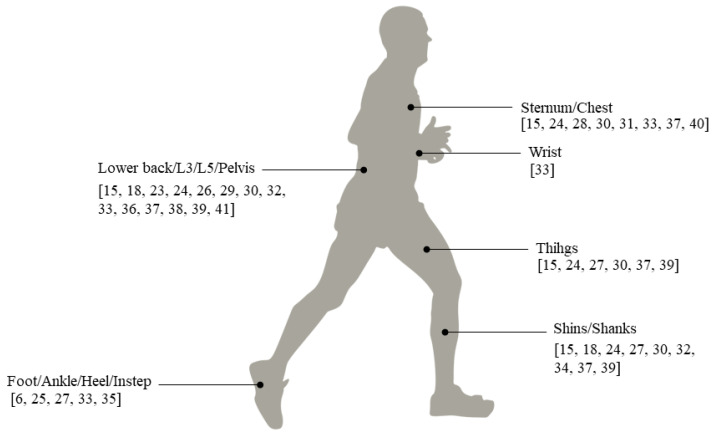
Sensors’ body locations and papers’ references.

All studies selected employed inertial sensors (some studies used other systems as well, but these systems were not considered in this work). The number of IMUs used ranged from 1–6. A large part of the studies used a single sensor, usually located on the back (lower back, L3 or L5 vertebrae) [24,27,30,37,39,42], or in some cases on the sternum [29,41], chest [32], or foot [6]. Three studies employed two IMUs, which were located on the same part of the body: feet [26], shins [35], and heels [36]. Studies that employed 3 IMUs [18,33], 5 IMUS [15,25,31,38,40], or 6 IMUs [28,34] combined different body positions as the thighs, shins, feet, shanks, back, ankles, pelvis, or sternum. The sensor’s body location is represented in the Figure 4.

#### 4.1.3. Motion Test

The most frequently repeated tests consisted of simply walking a fixed distance or time, normally between 3 and m 7 m or 0.5 min and 30 min [6,26,30,31,33,34,35,36,37,38,42]. Some studies realized more complex walking tests, in which the subject was performing another task such as counting (dual-task tests) [15,18,40].

Some studies used the IMUs to record data during the classical frailty test to extract the kinematic parameters. For example: Reference [24] analyzed the 30-s Chair Stand Test; Reference [41] captured the 10 m expanded Timed Up to Go test; Reference [25] used three physical assessments: TUG, Five-Times Sit To Stand, and quiet standing.

There were six studies that captured Daily Physical Activity (DPA) for long periods of time (days) to analyze the walking periods and parameters as total steps/day, walking percentage/day, or time in bed [15,27,29,31,32,39].

#### 4.1.4. Gait Parameters

Authors studied the significance of gait parameters by applying different statistical methods and a few of them classification methods based on artificial intelligence. Some of them used common statistical analysis tests such as ANOVA (ANalysis Of VAriance), t-Student test, or Spearman correlation, among others, [6,18,27,34,36,41]. A large number of the studies used conventional multivariable models: linear regression or logistic regression models [15,16,28,29,31,33,38,40]. Other methods such as partial least-squares discriminant analysis were applied by [37,39], and decision tree models were used by [24,32,42]. Moreover, there were studies that supported the use of new machine learning techniques such as neural network models [26,35] or support vector machine methods [25].

A large number of gait parameters were identified in the articles studied. These parameters are summarized in tables in order to analyze the number of times that each parameter was studied, as well as the number of times that a parameter was considered as *significant* to discriminate between patient groups. Table 4 and Table 5 contain the gait parameters related to frailty and fall risk, respectively, where the columns represent the articles included and a code number between 0 and 2, with the following meaning: 0 is *nonsignificant*; 1 is *included the study (more or less significant)*; 2 is *significant*. The last three columns are the total number of occurrences of each parameter (T), the significant cases (Sig, code = 2), and the nonsignificant cases (NSig, code = 0) of each gait parameter.

In the case of gait parameters related to frailty, they were grouped into: general parameters, the variability of general parameters, temporal parameters, spatial parameters, toe-specific parameters, specific gait phases’ speeds, daily physical activity parameters, and parameters from classical tests, as shown in Table 4.

From the fall risk studies, we identified a lower number of parameters, some of them repeated in frailty studies. They were divided into general and temporal parameters, variability, DPA parameters, and parameters related to trunk and corporal stability, as shown in Table 5.

Note that for a correct interpretation of the last two columns in Table 4 and Table 5, the ideal situation for a significant parameter is to find a high number in Sig and a zero or low number in NSig. The larger T, the larger the confidence level on that interpretation.

#### 4.1.5. Sensors’ Locations and Parameters

The relation between the gait parameters and the sensors’ body locations is an interesting aspect to know. Therefore, the estimated gait parameters for each sensor’s location and paper’s reference are summarized in Table 6. The sensors’ locations were divided into two groups, *single* and *combinations*. *Single locations* define that the inertial sensors were only located at one position: feet, or instep, or heels; chest or sternum; lower trunk (trunk, lumbar spine), or L3, or L5; shins. Some authors employed two or more body locations, *combinations*, which are represented by a character: A = shins/shanks + thighs + lower back/trunk; B = shanks + pelvis/L5; C = thighs + shins + feet; D = shins + thighs + L5 + sternum; E = wrist + ankles + L5 + sternum. The C, D, and E combinations were only employed by one author, while A was repeated by four authors and B by two authors.

## 5. Discussion

The main objective of this work was to identify the most relevant gait parameters to discriminate or classify frail levels and fall risk. The parameters were sorted and summarized in two tables, Table 7 and Table 8, which consider the number of times these parameters appeared in the literature and, further, the number of times when they were considered relevant for detection and classification purposes. These results were analyzed in depth in two subsections, one for parameters related to frailty and another for parameters related to fall risk.

### 5.1. Gait Parameters Related to Frailty

By analyzing the data in Table 4 and Table 7, we found that the most relevant temporal gait parameters related to frailty were: gait speed, double support time, stride time, step time, and the number of steps per day or the walking percentage/day, from daily physical activity parameters. All these parameters appeared several times (for or more), and all or most of them were considered as *significant* or able to discriminate between patient groups. Furthermore, they were never considered irrelevant in any article studied.

Frailty leads to physical deterioration and reduced mobility. For this reason, frailty patients show a longer double-support time (when both feet are standing on the floor) and a longer step and stride time than healthy persons. In addition, the gait speed is lower than in robust patients (this is the second Fried condition). Others symptoms of frailty such as the reduction of physical activity, low energy, or fatigue are represented in a lower number of steps/day or walking percentage/day, i.e, the amount of time that the patient spends walking every day.

The max number of steps per bout (PDA) or the kinematic parameters obtained from the Timed Up to Go test are relevant as well; although they only were included in two articles, but both considered them as important parameters. TUG test is widely used in clinical tests to diagnose frailty, so its manually use is validated. New approaches studied, monitoring this test using IMUs, seem to be successful and able to discriminate frailty states.

There are some parameters such as cadence or midswing speed that had contradictory results. In some cases, they were considered as relevant parameters, but in others, they were described as not significant. We can not obtain clear conclusions about this. Frailty can be associated with the gait or gait parameters’ variability or asymmetry, but by analyzing the stride length variability, stride time variability, speed variability, gait variability, or gait symmetry in the Table 7, there was not a clear outcome.

Several gait parameters were found in only one study, and they were considered important. This was the case of parameters such as toe-off angle, heel strike, or max toe. To measure them, it is necessary to locate the sensor on the foot, and only two articles used this body position. Furthermore, some articles developed classical tests as the 30-s Chair Test or Five-Times Sit to Stand to obtain kinematic parameters (as in the case of the TUG test), showing good results to discriminate patient groups. Time in bed and quantitative continuous walking are derived from DPA, coupled with the number of steps/day or walking percentage/day and the max number of steps per bout, supporting the measure of DPA using inertial sensors to assess frailty.

### 5.2. Gait Parameters Related to Fall Risk

In the case of fall risk, Table 5 and Table 8 indicate that trunk accelerations are important to predict or identify fall risk. This parameter appeared two times, and both considered it as as relevant; moreover, we can include the trunk stability, the trunk control, the dynamic stability, or the center of pressure deviations in this category (they appeared only one time, but all were considered significant in their respective studies, while neither as nonsignificant). From this, we can assume that when it comes to fall risk, the most relevant parameters are those related to stability and trunk movements.

Gait speed, stride length, and stride time have inconsistencies. By analyzing them in depth, we found that stride time, stride length, double-support time, gait symmetry, and gait speed, were considered as nonsignificant in [38]. The authors indicated that the results were inconsistent with previous reports, and this could be due to the nonfrail patient characteristics. The results suggested that balance (stability) and DPA parameters were predictive of fall risk in frail and prefrail adults. However, gait parameters were not able to discriminate between patient groups. In addition, they did not observe an increased risk of falling with increasing frailty status. In [33], gait speed and stride time also did not differ between patient groups. The relationship between frailty and the risk of falling is certain, and gait speed was considered as a relevant parameter in classic tests such as TUG, SPPB, or Fried’s scale.

Therefore, fall risk would also be related to a slower walking speed and stride time, but the discrepancies between different authors did not allow obtaining clear conclusions. In short, we can find a relationship between fall risk and trunk stability.

### 5.3. Limitations

The limitations of this study were due to the number of existing studies using IMUs for gait analysis in frailty patients, as well as the inconsistencies found in the results. Some gait parameters were considered relevant and not relevant in different studies.

Furthermore, not all papers estimated all parameters, just a subset of them. Therefore, this suggest that further studies with the frailty and fall risk population could be performed with a more complete range of estimated parameters.

## 6. Conclusions

In this work, a total of 22 articles were analyzed in order to obtain the most relevant gait parameters that can be measured with inertial sensors, and serve as indicators of patients’ frailty and fall risk. A literature review in the major databases was carried out, selecting the articles that complied with all the inclusion criteria such as older patient, frailty, and fall risk classification, inertial sensors, gait analysis, etc. Gait parameters used and studied in each article were marked and collected in tables, to analyze the importance of them.

In the case of frailty, gait speed, double-support time, stride time, and step time, and the number of steps per day or the walking percentage/day, from daily physical activity parameters, were the most relevant parameters. Stride length or kinematic parameters extracted from classic tests (such as the TUG test) were important as well.

For fall risk assessments, the study of the trunk stability and movements was crucial, being the most relevant parameter to classify between fallers and nonfallers. Other parameters, common with frailty, such as stride time, gait speed, or number of steps per day, can be successful as well.

We found inconsistency in some cases; for example, stride length was relevant in frailty, but not in the fall risk studies. However, this parameter is closely related to gait and age, which decreases with frailty and physical condition.

The use of IMUs is widely supported in the diagnosis of frailty or fall risk, and the literature reviewed showed satisfactory results for this type of application, assuming a new approach that would allow a more realistic gait analysis for the patients and even using them to obtain more parameters in the classical tests. This review highlighted the most relevant parameters that must be identified in frailty or prefrailty diagnosis applications using inertial sensors. It can serve as a guide for researchers and engineers who develop sensors and algorithms to analyze the gait in these patients.

As a future work, the authors of this paper consider it interesting to perform new studies with elderly people in order to assess the significance of most of the parameters tabulated in this review. In addition, the authors contemplate the study of classifying frailty patients by machine learning techniques and relevant gait parameters.

## Figures and Tables

**Table 1:** Fried’s frailty score and criteria. The five Fried’s conditions, their definitions, and possible values and the frailty score. The table was elaborated from [3].

Conditions	Definition	Value
1. Low physical activity level	Exercise hours or calories per week	0: No 1: Yes
2. Slowness	Slow walking speed (m/s)	0: No, 1: yes
3. Poor endurance and energy	Indicated by self-report of exhaustion.	0: No, 1: yes
4. Weakness	Grip strength in the lowest 20% at baseline	0: No, 1: yes
5. Shrinking	Unintentional weight loss	0: No, 1: Yes
**Frailty Score**	Sum of the value of the 5 conditions	0: Robust
		1–2: prefrail
		3–5: frail

**Table 2:** Summary of the reviews related to frailty or fall risk and gait analysis in the literature: author, year, patient’s condition, gait analysis technologies included in the article, number of papers included (M), relevant conclusions.

Author, Year	Condition *	Technology **	M	Relevant Conclusions
Vavasour, 2021 [10]	Frailty	Wearable sensors	29	Postural transitions, number of steps, and percentage of time in DPA and intensity of DPA together were the most frequently measured parameters followed closely by gait speed. All but one study demonstrated an association between PA and level of frailty. All reports of gait speed indicated correlation with frailty.
Patel, 2020 [16]	Falls	Inertial sensors	35	A single sensor located on the lower trunk (the most effective location) is enough to determine fall risk.
Zampogna, 2020 [19]	Others	Wearable sensors	62	Wireless sensors are a sensitive and objective tool for domestic measurement of control balance, postural dysfunction, gait disorders, or fall risk, providing data in free-living conditions and long-term monitoring. Most of the studies included used inertial devices.
Zhong, 2020 [11]	Falls	All	21	Parameters related to falls: gait speed, stride length, frequency, acceleration RMS, step-to-step consistency, autocorrelation and harmonic ratio.
Petraglia, 2019 [9]	Others	Inertial sensors	16	Good concordance between classic gait analysis methods and inertial sensors.
Montesinos, 2018 [17]	Falls	Inertial sensors	13	Lower back is the most common location. The most significant parameters related to fallers are: RMS acceleration mediolateral, No. of steps, time of TUG test, and step time.
Rucco, 2018 [12]	Falls	Wearable sensors	42	Accelerometers and gyroscopes are the most used sensors, while trunk is the most common location.
Mugueta-Aguinaba, 2017 [13]	Frailty	All	104	Supports the use of different technologies in frailty: prevention, care, diagnosis, and treatment.
Dasenbrock, 2016 [14]	Frailty	All	28	Parameters to diagnose frailty: stride length, double support time, gait speed, and cadence.
Taborri, 2016 [8]	-	All	32	Feet are the most useful location for accelerometers and gyroscopes in gait analysis.
Howcroft, 2013 [20]	Falls	Inertial sensors	40	Inertial sensors are promising sensors for fall risk assessment, and lower trunk is the most common location.
Schwenk, 2013 [21]	Frailty	All	11	Relevant gait parameters to discriminate between frail groups: gait speed, gait variability, cadence, step width variability, step length, and double-support time.

* Condition: the patient’s medical condition or pathology. The studies included patients with frailty (frailty), with fall risk (falls), or with other diseases (others). ** Technology: consists of the type of technology included in the studies: only inertial sensors (inertial sensors), all types of wearable devices (wearable sensors), and all types, both wearable and nonwearable (all).

**Table 3:** Summary of articles included: author, year; patients: medical condition, number of patients (N), and percentage of healthy patients; sensor and location: model, inertial axes used, number of IMUs (I), and location; tests. Note that L3 and L5 correspond to the 3rd and 5th Lumbar vertebra, respectively.

Author, Year	Patients	IMUs and Location	Motion Tests
Jung, 2021 [26]	Frailty N = 74 % healthy = 35	Xsens MVN 1 Gyr I = 2 Feet	7 m walking test
García-Villamil, 2021 [6]	Fall risk N = 21 % healthy = 47	G-STRIDE (custom-made) 3 Acc + 3 Gyr I = 1 Instep	30 min walking test
Del Din, 2020 [27]	Frailty N = 65 % healthy = 55	Axivity AX3 3 Acc I = 1 L5	Daily physical activity
Apsega, 2020 [28]	Frailty N = 133 % healthy = 23	Shimmer 3 Acc + 3 Gyr I = 6 thighs, shins, and feet	3 m TUG test
Padreep-Kumar, 2020 [29]	Frailty N = 126 % healthy = 34	PAMSys 1 Acc I = 1 Sternum	Daily physical activity
Porta, 2020 [30]	Fall risk N = 261 % healthy = 49	Xsens MTx 3 Acc + 3 Gyr I = 1 Back	3 m walking test
Jansen, 2019 [31]	Frailty N = 112 % healthy = 47	PAMSys 3 Acc I = 1 Sternum	Dayly physical activity
		LEGSys 3 Acc + 3 Gyr I = 5 Shank, thighs, and lower back	4.57 m normal walking test and 10 m fast walking test
Razjouyan, 2018 [32]	Frailty and mind N = 163 % healthy = 26	PAMSys 3 Acc I = 1 Chest	Daily physical activity
Bizovska, 2018 [33]	Fall risk N = 131 % healthy = 61	Trigno Wireless System 3 Acc I = 3 L5 and shanks	5 min walking test
Jehu, 2018 [34]	Fall risk and Parkinson’s N = 42 % healthy = 32	APDM 3 Acc I = 6 wrists, ankles, L5, and sternum	30 s walking test
Rahemi, 2018 [35]	Frailty N = 161 % healthy = 30	LEGsys 1 Gyr I = 2 Shins	4.57 m walking test
Howcroft, 2018 [18]	Fall risk N = 75 % healthy = 62	X16-1C 3 Acc I = 3 Lateral shanks and pelvis	7.62 m walking single-task and dual-task test
Ritt, 2017 [36]	Frailty N = 123 % healthy = 28	Shimmer 2R 3 Acc + 3 Gyr I = 2 Heels	Simple walking test
Millor, 2017 [24]	Frailty N = 718 % healthy = 27	Xsens MTx 3 Acc + 3 Gyr I = 1 L3	30-s Chair Stand Test and 3 m gait velocity test
Kikkert, 2017 [37]	Fall risk N = 61 % healthy = 59	Dynaport, MiniMod 2 Acc I = 1 L5	160 m walking test
Mohler, 2016 [38]	Frailty and fall risk N = 119 % healthy = 36	LEGSys 3 Acc + 3 Gyr I = 5 Shins, thighs, and lower back	4.57 m walking test
		PAMSys 3 Acc I = 1 Sternum	Daily physical activity
Ihlen, 2016 [39]	Frailty and fall risk N = 71 % healthy = 54	Dynaport Hybrid, McRoberts 3 Acc I = 1 Lower back	Daily physical activity
Thiede, 2016 [40]	Frailty and peripheral artery disease N = 17 % healthy = 47	LEGSys 3 Acc + 3 Gyr I = 5 Shins, thighs, and trunk	Normal walking, dual-task overground walk (counting 100 to 1), and fast walk (minimum of 25 steps)
Galan-Mercant, 2015 [41]	Frailty N = 30 % healthy = 53	Iphone4 3 Acc + 3 Gyr I = 1 Sternum	10 m expanded TUG test
Martinez-Ramirez, 2015 [42]	Frailty N = 718 % healthy = 45	MTx Xsens 3 Acc + 3 Gyr I = 1 Lumbar spine	3 m walking test
Schwenk, 2015 [15]	Frailty N=125 % healthy = 35	LEGSys 3 Acc + 3 Gyr I = 5 Shanks, thighs, and lower back	4.57 m walking single- and dual-task test
		PAMSys 3 Acc I = 1 Sternum	Daily physical activity
Greene, 2014 [25]	Frailty and fall risk N = 124 % healthy = 46	Shimmer 1 Acc + 1 Gyr I = 5 Shins, thigh, L5, and sternum	TUG, Five-Times Sit to Stand, and balance

**Table 4:** Gait parameters related to frailty. The parameters are rated from 0 to 2 for each article, where 0: nonsignificant, 1: studied and more or less significant, and 2: significant or discriminant. T: number of total Times that each parameter was included, Sig: number of times that each parameter was considered “Significant”, and NSig: number of times that a parameter was considered as “Nonsignificant” among frailty groups.

Parameter/Reference	[26]	[27]	[28]	[29]	[31]	[32]	[35]	[36]	[24]	[40]	[41]	[42]	[15]	[25]	T	Sig	NSig
**General Parameters**																	
Cadence (steps/min)			2									0			2	1	1
Stride Length (m)													2		1	1	0
Step Time (s)	2			2								1	1		4	2	0
Stride Time (s)	2		2	2									1		4	3	0
Gait Speed (m/s)			2		2				1	1		2	2		6	4	0
**Variability General Params.**																	
Stride Length Var (%)				0											1	0	1
Stride Time Var (%)				0											1	0	1
Gait Variability (%)				2											1	1	0
Gait Symmetry (%)				0											1	0	1
Speed Variability										2					1	1	0
Stride/Step Regularity												2			1	1	0
**Temporal Params.**																	
Stance Phase Time (s)	1		2												2	1	0
Swing Phase Time (s)	1		2												2	1	0
Double Support Time (s)	2		2							2			2		4	4	0
Propulsion Duration (s)							2								1	1	0
**Toe Specific Params.**																	
Toe-Off Angle (${}^{\circ }$)								2							1	1	0
Heal Strike Angle (${}^{\circ }$)								2							1	1	0
Max Toe (m)								2							1	1	0
**Specific Speeds**																	
Toe-Off Speed (${}^{\circ }$/s)							2								1	1	0
Midswing Speed (${}^{\circ }$/s)							2			0					2	1	1
Mid Stance Speed (${}^{\circ }$/s)							2								1	1	0
Propulsion Acceleration (${}^{\circ }$2/s)							2								1	1	0
Speed Norm (degree/s)							2								1	1	0
**Trunk-Derived Params.** RMS Trunk Acc.												2			1	1	0
THD Trunk Acc.												2			1	1	0
Trunk Sway										2					1	1	0
**DPA Params.**																	
No. of Steps/Day or Walking Percentage/Day		2		2	2	2							2		5	5	0
Time in bed						2									1	1	0
Max. No. of Steps/Bout				2	2										2	2	0
**Classic Test Params.**																	
TUG Kinematic Param.											2			2	2	2	0
30-s Chair Test k.p.									2						1	1	0
Five-Times Sit to Stand Acc.														2	1	1	0

**Table 5:** Gait parameters related to fall risk. The parameters are rated from 0 to 2 for each article, where 0: nonsignificant, 1: studied and more or less significant, and 2: significant or discriminant. T: number of total Times that each parameter is included, Sig: number of times that each parameter is considered “Significant”, and NSig: number of times that a parameter is considered as “Nonsignificant” between fall risk patients.

Parameter/Reference	[6]	[30]	[34]	[33]	[18]	[37]	[38]	[39]	T	Sig	NSig
**General and Temporal Params.**											
Stride Length (m)	2						0		2	1	1
Stride Time (s)				0		2	0		3	1	2
Gait Speed (m/s)	2			0		2	0		4	2	2
Cadence (steps/min)	1								1	1	0
Double Support Time (s)							0		1	0	0
Swing Phase Time (s)	1								1	0	0
**Variability Params.**											
Stride Length Var (%)	0	1							2	0	1
Stride Time Var (%)		1							1	0	0
Swing Time Var (%)	0								1	0	1
Gait Symmetry							0		1	0	1
**DPA Params. and Others**											
No. of Steps/Day or Walking Percentage/Day							2		1	1	0
Total Distance per Bout/Test	2								1	1	0
No. of Steps per Bout/Test	1								1	0	0
Total Time Walking per Bout/Test	0								1	0	1
**Trunk and Stability Params.**											
Trunk Accelerations						2		2	2	2	0
Trunk Stability				2					1	1	0
Trunk Control (k.p)			2						1	1	0
Center of Pressure (CoP) Deviations					2				1	1	0
Dynamic Stability					2				1	1	0

**Table 6:** Summary of sensor’s body location, gait parameters, and paper’s references. Single locations: sensors located at a single body position. Combination: sensors are located at two or more body positions, where: A = shins/shanks + thighs + lower back/trunk; B = shanks + pelvis/L5; C = thighs + shins + feet; D = shins + thighs + L5 + sternum; E = wrist + ankles + L5 + sternum.

		Single locations					Combinations		
**Parameter/Location**	Feet Instep Heels	Chest Sternum	Trunk L3 L5	Shins	A	B	C	D	E
**General Parameters**									
Cadence (steps/min)	[6]		[42]				[28]		
Stride Length (m)	[6]				[15,38]				
Step Time (s)	[26]	[29]	[42]		[15]				
Stride Time (s)	[26]	[29]	[37]		[15,38]	[33]	[28]		
Gait Speed (m/s)	[6]		[24,37,42]		[15,31,38,40]	[33]	[28]		
**Variability Params.**									
Stride Length Var (%)	[6]	[29]	[30]						
Stride Time Var (%)		[29]	[30]						
Gait Variability (%)		[29]							
Gait Symmetry (%)		[29]			[38]				
Speed Variability					[40]				
Stride/Step Regularity			[42]						
Swing Time Var (%)	[6]								
**Temporal Params.**									
Stance Phase Time (s)	[26]						[28]		
Swing Phase Time (s)	[6,26]						[28]		
Double Support Time (s)	[26]				[15,38,40]		[28]		
Propulsion Duration (s)				[35]					
**Toe Specific Params.**									
Toe-Off Angle (${}^{\circ }$)	[36]								
Heal Strike Angle (${}^{\circ }$)	[36]								
Max Toe (m)	[36]								
**Specific Speeds**									
Toe-Off Speed (${}^{\circ }$/s)				[35]					
Mid-Swing Speed (${}^{\circ }$/s)				[35]	[40]				
Mid Stance Speed (${}^{\circ }$/s)				[35]					
Propulsion Acceleration (${}^{\circ }$2/s)				[35]					
Speed Norm (${}^{\circ }$/s)				[35]					
**DPA Params and Others.**									
No. of Steps/Day or Walking Percentage/Day		[15,29,31,32,38]	[27]						
Time in Bed		[32]							
Max. No. of Steps/Bout		[29,31]							
Total Distance per Bout/Test	[6]								
No. of Steps per Bout/Test	[6]								
Total Time Walking per Bout/Test	[6]								
**Classic Tests Params.**									
TUG Kinematic Param.		[41]						[25]	
30-s Chair Test k.p.	[24]								
Five-Times Sit to Stand Acc.								[25]	
**Trunk and Stability Params.**									
Trunk Accelerations			[37,39]						
Trunk Stability						[33]			
Trunk Control (k.p)									[34]
Center of Pressure (CoP) Deviations						[18]			
Dynamic Stability						[18]			
RMS Trunk Acc.			[42]						
THD Trunk Acc.			[42]						
Trunk Sway					[40]				

**Table 7:** Gait parameters related to frailty sorted by importance (total times and significance). R: Ranking, T: number of total Times that each parameter was included, Sig: number of times that each parameter is considered Significant, and NSig: number of times that a parameter is considered as “Nonsignificant” among frailty patients.

R	Parameter	T	Sig	NSig
1	No. of Steps/Day or Walking Percentage/Day	5	5	0
2	Gait Speed (m/s)	6	4	0
3	Double Support Time (s)	4	4	0
4	Stride Time (s)	4	3	0
5	Step Time (s)	4	2	0
6	TUG Kinematic Param and Max Number of Steps per Bout	2	2	0
7	Stance Phase Time (s) and Swing Phase Time (s)	2	1	0
8	Cadence (steps/min) and Midswing Speed (${}^{\circ }$/s)	2	1	1
9	Stride Length (m), Gait Variability (%), Speed Variability, Stride/Step Regularity, Propulsion Duration (s), Toe-Off Angle (${}^{\circ }$), Heal Strike Angle (${}^{\circ }$), Max Toe (m), Toe-Off Speed (${}^{\circ }$/s), Mid Stance Speed (${}^{\circ }$/s), Propulsion Acceleration (${}^{\circ }$2/s), Speed Norm (${}^{\circ }$/s), Root-Mean-Squared (RMS) and Total Harmonic Distortion (THD) from Trunk Accelerations, Trunk Sway, Time in Bed, 30-s Chair Test Kinematic Parameters, Five-Times Sit to Stand Accelerations	1	1	0
10	Stride Length Var (%), Stride Width Var (%), and Gait Symmetry (%)	1	0	1

**Table 8:** Gait parameters related to fall risk sorted by importance (total times and significance). R: Ranking, T: number of total Times that each parameter is included, Sig: number of times that each parameter is considered “Significant”, and NSig: number of times that a parameter is considered as “Nonsignificant” among fall risk patients. Symbol “*” marks the inconsistencies.

R	Parameter	T	Sig	NSig
1	Trunk Accelerations	2	2	0
2	Gait Speed (m/s)	4	2	2 *
3	Stride Time (s)	3	1	2 *
4	Stride Length (m)	2	1	1 *
5	Cadence (steps/min), Total Distance per Bout/Test, Trunk Stability, Trunk Control (Kinematic Parameters), Center of Pressure (CoP) Deviations, Dynamic Stability	1	1	0
6	Swing Phase Time (s), Number of Steps per Bout/Test, and Stride Time Var (%)	1	0	0
7	Stride Length Var (%)	2	0	1
8	Double-Support Time (s), Swing Time Var (%), Gait Symmetry, Total Time Walking per Bout/Test	1	0	1

## Abbreviations

The following abbreviations are used in this manuscript:

IMU	Inertial Measurement Unit
DPA	Daily Physical Activity
TUG	Timed Up and Go test
SPPB	Short Physical Performance Battery
30-s CST	30-second Chair Stand Test
FTSS	Five-Times Sit to Stand
GC	Gait Cycle
HS	Heel Strike
FFL	Foot Flat
TO	Toe-Off
DS	Double-Support
HL	Heel Lift
SL	Strike Length
GCT	Gait Cycle Time
L3	Third Lumbar vertebra
L5	Fifth Lumbar vertebra
